# Assessing the effectiveness of machine learning and deep learning in differentiating neuroimmunological diseases: a systematic review and meta-analysis

**DOI:** 10.3389/fneur.2025.1579206

**Published:** 2026-01-12

**Authors:** David Petrosian, Natasa Giedraitiene, Rasa Kizlaitiene, Dalius Jatuzis, Gintaras Kaubrys, Mantas Vaisvilas

**Affiliations:** 1 Faculty of Medicine, Vilnius University, Vilnius, Lithuania; 2 Clinic of Neurology and Neurosurgery, Institute of Clinical Medicine, Vilnius University, Vilnius, Lithuania

**Keywords:** artificial intelligence, deep learning, differential diagnosis, machine learning, neuroimmunology

## Abstract

**Objective:**

The differential diagnosis of neuroimmunological disorders remains a significant challenge in clinical practice, even with advancements in diagnostic techniques. Recently, the use of artificial intelligence (AI) for diagnosing and distinguishing between various neuroimmunological disorders has gained traction. Our objective was to conduct a systematic review and meta-analysis to evaluate the diagnostic performance of Machine Learning (ML) and Deep Learning (DL) techniques in differentiating these disorders. We aimed to identify the most effective approaches, compare their diagnostic outcomes, and offer recommendations for improving their applicability across multiple clinical centers and for future research.

**Methods:**

Following the PRISMA 2020 guidelines, a systematic search in PubMed and Web of Science was conducted to identify relevant articles published between 2000 and 2024 that fell within the scope of our research. QUADAS-2 tool was assessed to evaluate the risk of bias and applicability concerns. The performed meta-analysis allowed us to estimate the overall accuracy, sensitivity, and specificity of the developed models providing quantitative insights from this analysis.

**Results:**

Of 4,470 articles identified, 19 met inclusion criteria: 9 (47.4%) used ML and 10 (52.6%) used DL. Most models relied on MRI data to differentiate multiple sclerosis from neuromyelitis optica spectrum disorders. Pooled accuracy, sensitivity, and specificity were 0.87, 0.86, and 0.84, respectively. Substantial heterogeneity was observed, which decreased in a sensitivity analysis excluding larger-sample studies and varied between ML and DL models, with ML showing lower heterogeneity.

**Conclusion:**

New AI tools, primarily utilizing MRI data, are emerging and demonstrate the potential to differentiate between various neuroimmunological disorders. While most neuroimmunological conditions have accessible antibody tests with strong diagnostic performance, AI efforts should concentrate on seronegative diseases. This approach should incorporate clinical and epidemiological data into diagnostic algorithms for improved accuracy.

## 1 Introduction

Differential diagnosis of neuroimmunological disorders remains challenging in clinical practice despite evolving diagnostic techniques (1–3). Application of artificial intelligence (AI) to diagnose (4) and differentiate between multiple sclerosis (MS), neuromyelitis optica spectrum disorders (NMOSD), myelin oligodendrocyte glycoprotein antibody-associated disease (MOGAD), and autoimmune encephalitis (AE) have been increasingly exploited. On the one hand, AI techniques may benefit standard clinical care by processing large amounts of information, including clinical data and radiological images, to identify patterns undetectable by conventional means (5) aiding decision-making and reducing the risk of human error (6).

On the other hand, despite the benefits mentioned, only a small proportion of AI tools are applied internationally (7). Similarly, the use of AI tools in neuroimmunological disorders is limited, with only a few studies published to date (8). To assess advancements in AI techniques within the field of neuroimmunology, we conducted a systematic review and meta-analysis to evaluate the application of ML and DL techniques in differentiating neuroimmunological diseases. Our goals include identifying commonly used approaches, analyzing their diagnostic performance, and providing recommendations to enhance their applicability across various clinical centers and future research.

A previous systematic review and meta-analysis focused primarily on MS versus NMOSD and reported substantial heterogeneity (9). Our study extends these findings by examining contributors to heterogeneity, such as study size, dataset composition, and model architecture, and emphasizes the need for improved methodological rigor.

## 2 Material and methods

### 2.1 Information sources and search

A systematic search was conducted to select articles that fall within the scope of our research. We reviewed publications in PubMed and Web of Science databases published from 2000 to 2024. The temporal search range was preregistered to ensure comprehensive coverage of earlier literature. The search strategy that was applied: (((((((((Multiple sclerosis) OR (Autoimmune encephalitis)) OR (Neuromyelitis optica)) OR (NMOSD)) OR (NMO)) OR (Devic's disease)) OR (Myelin oligodendrocyte glycoprotein)) OR (MOG)) OR (MOGAD)) AND ((((Artificial intelligence) OR (Machine Learning)) OR (Deep Learning)) OR (Neural network)). Search results were included or excluded in the final analysis based on the criteria shown below, accordingly.

### 2.2 Eligibility criteria

Studies were considered eligible for inclusion if they met all of the following criteria: (1) investigated the differentiation between neuroimmunological diseases (e.g., MS, NMOSD, AE, MOGAD), (2) utilized Machine Learning or Deep Learning techniques for classification or diagnostic purposes, (3) involved human subjects, (4) were available as full-text articles, (5) were published from the year 2000 onward, and (6) were written in English.

### 2.3 Screening

Two reviewers (D.P. and M.V.) independently performed the screening process. After excluding articles based on the titles and abstracts that are out of our scope, the rest were sought for retrieval as potentially eligible and were assessed full text.

### 2.4 Data collection and items

Two reviewers (D.P. and M.V.) independently performed data extraction to ensure accuracy and reduce bias. Any discrepancies between reviewers were resolved through discussion, and, if necessary, a third reviewer (N.G.) was consulted to reach consensus. Data was extracted into the spreadsheet we created to include the relevant data. Further metadata was extracted: 1. first author; 2. year of article; 3. neuroimmunological diseases; 4. objective of the study; 5. used parameters, e.g. clinical data, MRI images; 6. data source; 7. AI technique, e.g. Machine Learning or Deep Learning; 8. model performance, e.g. accuracy, specificity, sensitivity, area under the curve (AUC).

### 2.5 Data synthesis

Extracted relevant data was categorized and tabulated to facilitate a comprehensive analysis. Categorization was primarily based on AI techniques, distinguishing whether the classification task was performed with Machine Learning or Deep Learning models. By conducting a meta-analysis we chose a random-effects model due to variations in parameter characteristics, patient populations (data sources), and AI algorithms. *I*^2^ and τ statistics were used to assess the degree of data heterogeneity (10). The meta-analysis was conducted using the meta package in R 4.2.2 (11).

Among the included studies, MRI-based MS versus NMOSD comparisons represented the largest and most methodologically comparable group, and were therefore included in the quantitative meta-analysis. For studies reporting multiple ML or DL models, the model with the highest reported performance metrics was selected for inclusion in the quantitative meta-analysis. Studies using imaging modalities other than MRI or investigating neuroimmunological diseases other than MS versus NMOSD were planned to be synthesized narratively if they were few in number or methodologically heterogeneous, rather than included in the quantitative meta-analysis.

For studies that did not provide full 2 × 2 tables, counts were reconstructed from the reported sensitivity, specificity, accuracy, and corresponding sample sizes, and these reconstructed counts were used for the meta-analysis. Meta-analyses of sensitivity, specificity, and accuracy were performed using the metaprop() function. A univariate random-effects model with inverse-variance weighting was applied, and logit transformation (sm = “PLOGIT”) was used to stabilize variances. In the meta-analysis, all counts—both reported and reconstructed—were sufficient for analysis without requiring a continuity correction. Univariate models were chosen rather than bivariate or HSROC models because the number of included studies was limited and several studies did not report complete 2 × 2 tables, making estimation of between-study correlation unreliable.

Publication bias was assessed using Deeks' funnel plot asymmetry test. A *p*-value > 0.05 was considered to indicate no significant small-study effects.

### 2.6 Quality assessment and risk of bias

Risk of bias and applicability were assessed using the QUADAS-2 tool (12), which evaluates four domains: patient selection, index test, reference standard, and flow and timing. Two reviewers (D.P. and M.V.) independently conducted the assessments. Discrepancies were resolved through discussion and consensus, and overall inter-reviewer agreement was high. Each domain was rated as low, high, or unclear risk based on standard criteria. High risk was assigned for patient selection with non-transparent or non-representative sampling; index tests lacking sufficient methodological detail; reference standards without clearly defined diagnostic criteria; and flow or timing concerns regarding the application of the diagnostic process.

## 3 Results

### 3.1 Study selection and characteristics

**Figure 1:**
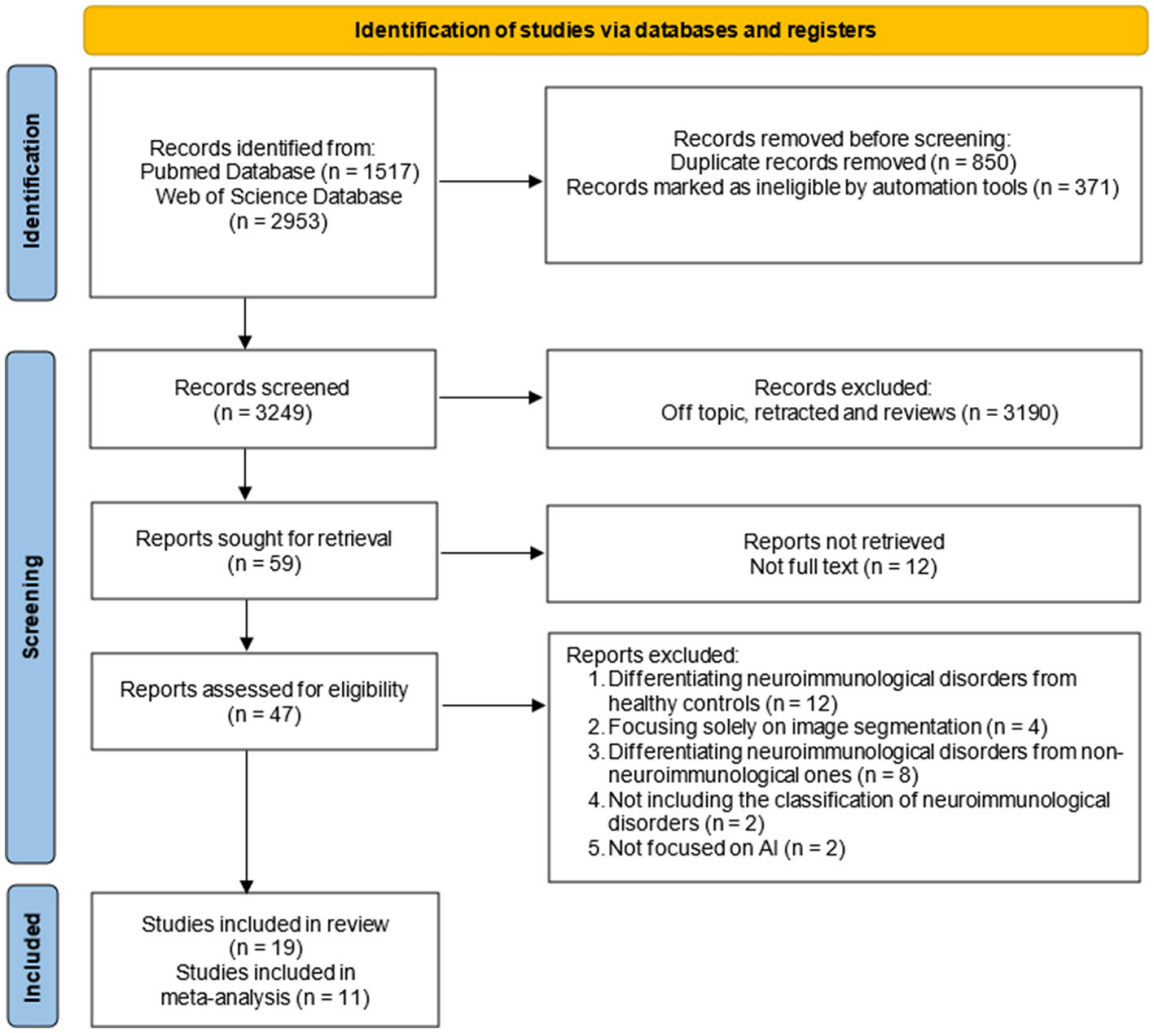
PRISMA flow diagram depicting the flow of study selection.

Our search strategy identified a total of 4,470 publications. After screening, results from 19 articles met the inclusion criteria and were included in the systematic review. Figure 1 presents a diagram depicting the flow of study selection.

All the studies included in this systematic review and meta-analysis were published between 2020 and 2024. Nine publications (47.4%) implemented ML algorithms, while ten (52.6%) utilized DL techniques.

As shown in Table 1, among the articles applying ML, the most common application (*n* = 5) was differentiating between MS and NMOSD. Other studies developed AI techniques mainly between antibody-associated demyelinating disease (NMOSD and MOGAD, respectively).

**Table 1:** Studies differentiating neuroimmunological diseases using machine learning.

**Study**	**Diseases**	**Data source**	**Model**	**Imaging modality**	**Parameters**	**Performance**	**Training / validation set**	**Test set**
El Khoury et al. (24)	MS vs. NMOSD	60 MS, 60 NMOSD	Random forest	–	Fourier-transform infrared spectra of serum samples	AUC: 100%, Sensitivity: 100%, Specificity: 100%, Precision: 100%	108	12
Yan et al. (25)	MS vs. NMOSD	47 MS, 36 NMOSD	Logistic regression	MRI	Brain radiomics signatures and demographic information	Combined model: AUC of 0.927 (95% CI: 0.871–0.984), Demographic information-only model: AUC of 0.733 (95% CI: 0.639–0.818), Sensitivity 0.511, Specificity 0.861, Accuracy 0.663, Radiomics-only model: AUC of 0.902 (95% CI: 0.840–0.955), Sensitivity 0.851, Specificity 0.889, Accuracy 0.867	83	–
Clarke et al. (26)	MS vs. NMOSD	100 MS, 66 NMOSD	Decision tree	MRI	Brain, spine, orbits T1, T2, FLAIR sequences	TP: 60, FP: 4, TN: 96, FN: 6, TP rate: 0.929, FP rate: 0.060, Precision 0.939, F-measure: 0.934, AUC: 0.935	–	–
Huang et al. (27)	MS vs. NMOSD	78 MS, 38 NMOSD	Random forest	MRI	Brain radiomic features (extracted from T1-MPRAGE and T2 sequences), clinical features	Multi-parametric MRI: AUC 0.902 ± 0.027, Sensitivity 0.873 ± 0.083, Specificity 0.869 ± 0.051, Accuracy 0.871 ± 0.044	86	30
Gharaibeh et al. (28)	MS vs. NMOSD	424 MS, 261 NMOSD	KNN (VGG16, VGG19, InceptionV3 for feature extraction)	MRI	Brain features extracted from FLAIR and T2W sequences	VGG16: KNN: Precision 0.98, Recall 0.99, F1-score 0.99, Accuracy 0.99 VGG19: KNN: Precision 0.96, Recall 0.98, F1-score 0.97, Accuracy 0.97 InceptionV3: KNN: Precision 0.92, Recall 0.95, F1-score 0.93, Accuracy 0.93	548	137
Ciftci Kavaklioglu et al. (29)	MS vs. (NMOSD and MOGAD)	57 MS, 11 NMOSD, 27 MOGAD	Random forest	OCT	OCT features	Accuracy: 0.68, Sensitivity: 0.69, Specificity: 0.67, AUC: 0.73	76	19
Luo et al. (30)	MS vs. (NMOSD and MOGAD); NMOSD vs. MOGAD	63 MS, 87 NMOSD, 45 MOGAD	Random forest, logistic regression	MRI	Brain radiomics and spatial distribution features of brain lesions extracted from T1, T2-FLAIR sequences	1. MS vs. (NMOSD and MOGAD) Joint model: AUC 0.927, Accuracy 0.863, Sensitivity 0.858, Specificity 0.868 2. MOGAD vs NMOSD Joint model: AUC 0.871, Accuracy 0.805, Sensitivity 0.808, Specificity 0.805	(1) 195 (2) 132	–
Ding et al. (31)	MOGAD vs. non-MOGAD	66 MOGAD, 66 non-MOGAD	Support vector machine	MRI	Radiomic features extracted from T1WI, T2WI, T2W-FLAIR, DWI sequences	Internal test set (AUC 0.844, Accuracy 83.33%, Sensitivity 85.71%, Specificity 81.25%) External test set (AUC 0.846, Accuracy 80.65%, Sensitivity 93.75%, Specificity 66.67%)	101	31
Wei et al. (32)	ADEM vs. MOGAD	49 ADEM, 21 MOGAD	Multilayer perceptron, support vector machine	MRI	Brain radiomic features extracted from FLAIR sequence	0–6y Female: MLP: Accuracy 0.784, F1 0.556, Specificity 0.774, Sensitivity 0.833, AUC 0.903 0–6y Male: SVM: Accuracy: 0.805, F1 0.638, Specificity 0.821, Sensitivity 0.750, AUC 0.890 0–14y Female: SVM: Accuracy: 0.891, F1 0.759, Specificity 0.885, Sensitivity 0.917, AUC 0.981 0–6y Male: SVM: Accuracy: 0.971, F1 0.857, Specificity 1.000, Sensitivity 0.750, AUC 0.992	70	–

MS, Multiple Sclerosis; NMOSD, Neuromyelitis Optica Spectrum Disorder; MOGAD, Myelin Oligodendrocyte Glycoprotein Antibody Disease; ADEM, Acute Disseminated Encephalomyelitis; F1, F1 Score; MRI, Magnetic resonance imaging; OCT, Optical Coherence Tomography.

Similarly, most studies using DL applied neural networks to distinguish MS vs NMOSD. Other studies heterogeneously used models to differentiate between antibody-associated nervous system disorders. Table 2 lists the studies employing DL techniques.

**Table 2:** Studies differentiating neuroimmunological diseases using deep learning.

**Study**	**Diseases**	**Data source**	**Model**	**Imaging modality**	**Parameters**	**Performance**	**Training /validation set**	**Test set**
Cacciaguerra et al. (33)	MS vs. NMOSD	95 MS, 85 NMOSD	ResNet	MRI	Brain T2- and T1-weighted sequences	Accuracy: 0.95, MAE of 0.21, and MSE of 0.07	180	–
Seok et al. (34)	MS vs. NMOSD	86 MS, 70 NMOSD	ResNet18	MRI	Brain FLAIR sequences	Accuracy: 76.1%, Sensitivity: 77.3%, Specificity: 74.8%, PPV: 76.9%, NPV: 78.6%, AUC: 0.85	156	–
Kim et al. (35)	MS vs. NMOSD	213 MS, 125 NMOSD	ResNeXt	MRI	Brain 2D FLAIR sequences, clinical data	Accuracy: 71.1%, Sensitivity: 87.8%, Specificity, 61.6%, AUC:0.82	203	135
Hagiwara et al. (36)	MS vs. NMOSD	35 MS, 18 NMOSD	SqueezeNet	MRI	Brain multi-dynamic multi-echo sequence	AUC:0.859. MS Sensitivity: 80.0%, NMOSD Sensitivity: 83.3%. Accuracy: 81.1%	53	–
Zhuo et al. (37)	MS vs. NMOSD	134 MS, 186 NMOSD	MultiResUNet, DenseNet121	MRI	Spine T2-weighted sequence	Accuracy: 79.5%, Sensitivity: 80.0%, Specificity: 78.8%, PPV: 83.7%, NPV: 74.3%, Precision: 83.7%, Recall: 80.0%, AUC:0.85	242	78
Wang et al. (38)	MS vs. NMOSD	41 NMOSD, 47 MS	Pre-trained ResNet18	MRI	Brain T2-FLAIR sequence	Accuracy: 0.750, Sensitivity: 0.707, Specificity: 0.759	88	–
Huang et al. (39)	MS vs. NMOSD	69 MS, 62 NMOSD^†^	ResNet	MRI	Brain T2-FLAIR sequence	Accuracy: 92.16%, Sensitivity: 95.60%, Specificity: 92.60%, AUC: 96.33%	131	–
Huang et al. (40)	MS vs. (MOGAD and NMOSD) NMOSD vs. (MS and MOGAD) MOGAD vs. (MS and NMOSD)	67 MS, 162 NMOSD, 61 MOGAD	MIL-CoaT	MRI	Brain T2WI, brain T2-FLAIR, cervicothoracic T2WI, and thoracolumbar T2WI sequences	MS vs. (MOGAD and NMOSD) Brain MRI (AUC:0.936, Accuracy: 88.9%, Sensitivity: 78.6%, Specificity: 92.5%, PPV: 78.6%, NPV: 92.5%, F1: 0.786) NMOSD vs. (MS and MOGAD) Combined brain and spinal cord MRI (AUC: 0.942, Accuracy: 88.1%, Sensitivity: 87.9%, Specificity: 88.5%, PPV: 90.6%, NPV: 85.2%, F1: 0.892) MOGAD vs. (MS and NMOSD) Combined brain and spinal cord MRI (AUC: 0.803, Accuracy: 72.9%, Sensitivity: 83.3%, Specificity: 70.2%, PPV: 41.7%, NPV: 94.3%, F1: 0.556)	231	59
Zhou et al. (41)	NMOSD vs. ADEM	16 NMOSD, 174 ADEM	M-DDC	MRI	Brain MRI images	Precision: 96.96%, Recall: 96.96%, Accuracy: 99.19%, AUC: 96.66, Fβ: 96.96%	152	38
Pan et al. (42)	AE(LGI1) vs. AE(GABAB)	64 AE(LGI1), 17 AE(GABAB)	ResNet18	PET/CT	Brain PET/CT images	AUC: 0.98, Accuracy: 96.30%, Sensitivity: 94.12%, Specificity: 96.88%	81	–

MS, Multiple Sclerosis; NMOSD, Neuromyelitis Optica Spectrum Disorder; MOGAD, Myelin Oligodendrocyte Glycoprotein Antibody Disease; ADEM, Acute Disseminated Encephalomyelitis; and AE, Autoimmune Encephalitis associated with Leucine-Rich Glioma-Inactivated 1 (LGI1) or GABAB, Gamma-Aminobutyric Acid Receptor B; MAE, Mean Absolute Error; MSE, Mean Squared Error; PPV, Positive Predictive Value; NPV, Negative Predictive Value; F1, F1 Score; Fβ, F-beta Score; MRI, Magnetic Resonance Imaging; PET/CT, Positron Emission Tomography/Computed Tomography.

^†^Number of MRI image samples.

Reporting of seronegative patients was limited: only a small subset of the included studies explicitly stated whether seronegative cases were part of their cohorts.

### 3.2 Risk of bias and applicability concerns

The quality assessment using QUADAS-2 revealed several methodological limitations across the included studies. Many models were developed using single-center datasets with relatively small sample sizes, increasing the potential for bias and limiting generalizability. Case selection procedures were often insufficiently described, making it unclear whether participants were enrolled consecutively or randomly, and whether study populations were representative of the broader clinical cohorts. Limited reporting of performance metrics and validation methods, such as cross-validation or external testing, further raised concerns regarding the robustness of the reported diagnostic performance. Additionally, a few studies relied on parameters not routinely available in standard clinical settings (e.g., PET/CT), which may restrict reproducibility and wider applicability. Supplementary Table 2 provides full QUADAS-2 ratings for all included studies, summarizing domain-level risk-of-bias and applicability assessments.

**Figure 2:**
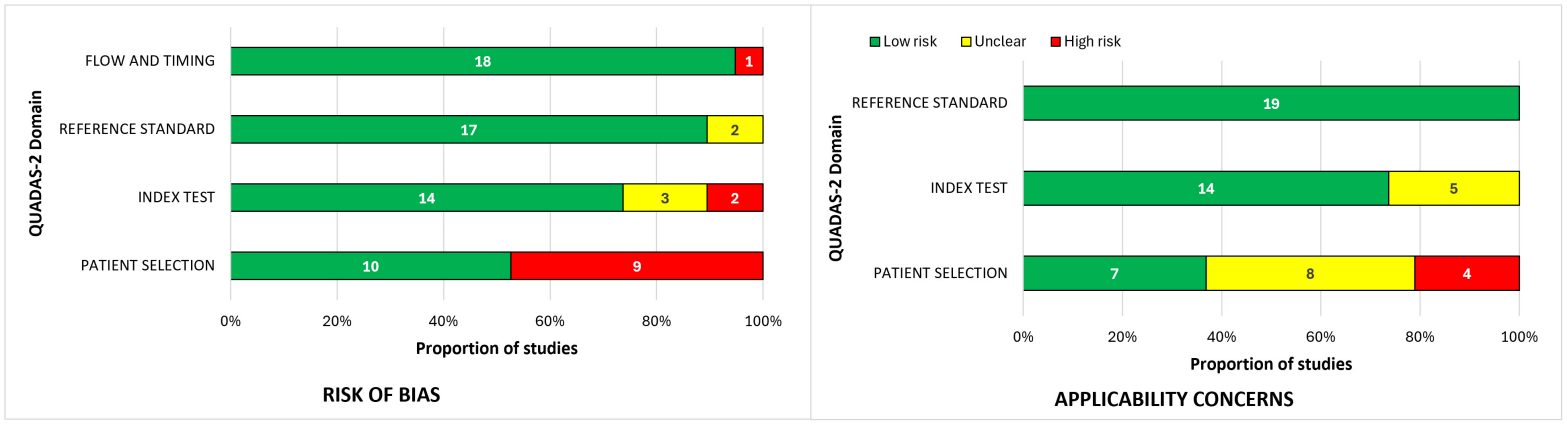
The proportion of studies assessed as having high, low, or unclear risk of bias and applicability concerns.

The highest risk of bias was observed in the patient selection domain (*n* = 9, 47.4% of all studies). A high risk of bias was assessed for models based on a limited number of disease subtypes, such as relapsing-remitting multiple sclerosis (RRMS) or seropositive NMOSD, not including others. The lack of transparency of data inclusion also increases the risk, raising concerns about the further applicability of such models. In contrast, index test, reference standard, and flow and timing domains had low risk in 73.7%, 89.5%, and 94.7% of studies, respectively. Despite the quite significant risk of bias in the patient selection domain, in terms of applicability, most studies were rated as less high risk, as illustrated in Figure 2.

### 3.3 Meta-analysis

To perform a meta-analysis, we estimated the pooled accuracy, sensitivity, and specificity to provide a comprehensive understanding of the diagnostic performance of ML and DL models in differentiating neuroimmunological diseases. We included 11 studies that investigated the differentiation between MS and NMOSD based on brain and/or spinal MRI data. Studies using other parameters, such as optical coherence tomography (OCT) or serum samples, were excluded, as including them would increase heterogeneity, especially given that only two such studies were available.

**Figure 3:**
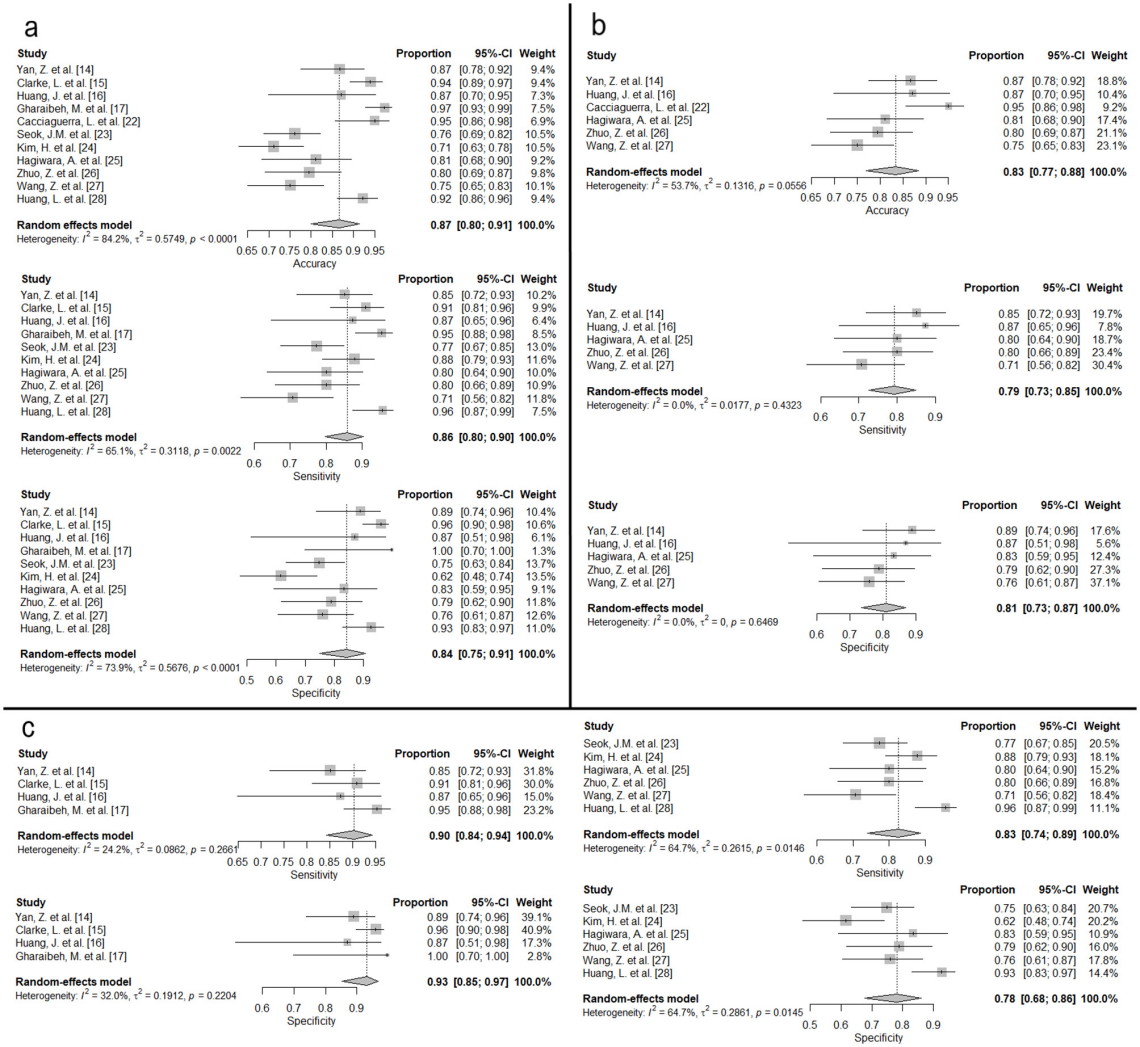
Forest plots of pooled diagnostic performance. **(a)** All included studies. **(b)** Studies with sample sizes < 100. **(c)** Studies stratified by model type (ML and DL).

After removing outlier studies—specifically, studies that used modalities other than MRI or investigated neuroimmunological diseases outside MS vs. NMOSD, as their outcomes were not directly comparable—we performed a random-effects meta-analysis to estimate pooled diagnostic performance (see Supplementary Table 1 for the full list of included and excluded studies). Models classifying between MS and NMOSD achieved a pooled accuracy of 0.87, indicating strong overall performance. The pooled sensitivity and specificity were 0.86 and 0.84, respectively. Substantial heterogeneity was found across studies for accuracy (*I*^2^ = 84.2%) and specificity (*I*^2^ = 73.9%), while heterogeneity for sensitivity was moderate (*I*^2^ = 65.1%; Figure 3a).

To account for heterogeneity, we conducted a secondary analysis by excluding studies with sample sizes greater than 100, thereby including only smaller sample studies. As illustrated in Figure 3b, the pooled accuracy, sensitivity, and specificity in this subset were 0.83, 0.79, and 0.81, respectively. Notably, heterogeneity was markedly reduced in this analysis (accuracy: *I*^2^ = 53.7%; sensitivity: *I*^2^ = 0.0%; specificity: *I*^2^ = 0.0%).

We also performed subgroup analyses by model type (Figure 3c). In the ML group, the pooled sensitivity was 0.90 and the pooled specificity was 0.93. Heterogeneity was low to moderate (sensitivity: *I*^2^ = 24.2%; specificity: *I*^2^ = 32.0%). In the DL group, pooled sensitivity and specificity were 0.83 and 0.78, respectively. Heterogeneity was higher in this group (sensitivity: *I*^2^ = 64.7%; specificity: *I*^2^ = 64.7%).

### 3.4 Publication bias

**Figure 4:**
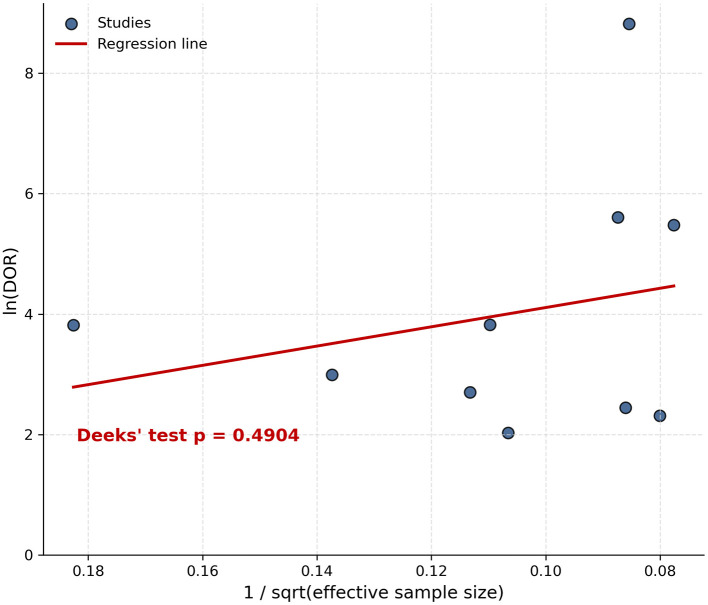
Funnel plot assessing publication bias in the included diagnostic accuracy studies. Deeks' asymmetry test showed *p* > 0.05.

The visual inspection of the funnel plot revealed a symmetrical distribution of the included studies around the regression line, suggesting the absence of small-study effects (Figure 4). In addition, Deeks' asymmetry test yielded a non-significant result (*p* = 0.4904), indicating no statistically significant evidence of publication bias. These findings suggest that the likelihood of publication bias influencing the pooled diagnostic accuracy estimates is low.

## 4 Discussion

In this review, we synthesized current evidence on AI applications for differentiating neuroimmunological disorders and performed a meta-analysis to evaluate the diagnostic performance of these models. Although individual studies frequently reported solid diagnostic accuracy, their results varied substantially, reflecting differences in study design, dataset characteristics, and modeling approaches.

Our meta-analysis demonstrated strong overall performance of AI-based models in distinguishing MS from NMOSD, with pooled accuracy, sensitivity, and specificity of 0.87, 0.86, and 0.84, respectively. Heterogeneity was substantial for accuracy and specificity and moderate for sensitivity; however, it decreased markedly after excluding large-sample studies, indicating that dataset size contributed significantly to variability. Subgroup analyses showed that ML models achieved higher pooled sensitivity (0.90) and specificity (0.93)—with lower heterogeneity—compared with DL models (0.83 and 0.78). While ML models showed higher pooled sensitivity and specificity than DL models, these comparisons are exploratory and should not be interpreted as definitive evidence of superiority. These ML–DL comparisons should be interpreted cautiously, as DL generally requires larger and more diverse datasets, which were often lacking in the included studies. Overall, results suggest that differences in dataset composition, sample size, and model architecture influenced the robustness of pooled estimates.

Methodological limitations identified through risk-of-bias assessment—particularly single-center design and unclear case selection—may affect the reliability and generalizability of reported model performance. Studies with narrowly defined or non-random samples can inflate accuracy estimates because models are trained on relatively homogeneous populations that may not reflect real-world clinical variability. In contrast, the greater heterogeneity observed in large-sample studies and DL models likely reflects increased variability in patient characteristics and technical factors, such as MRI acquisition protocols, preprocessing, and network design. Despite these sources of variation, Deeks' funnel plot asymmetry test did not indicate publication bias.

Limitations in the imaging modalities used across studies may further influence diagnostic performance. Most studies relied solely on cranial MRI, although spinal MRI provides critical diagnostic information—such as longitudinally extensive transverse myelitis or conus lesions—that strongly supports antibody-mediated demyelinating diseases. In contrast, optic nerve involvement, common across multiple neuroimmunological disorders, may be less clearly characterized on cranial imaging (13, 14). Comprehensive neuraxial imaging and analysis of larger, clinically representative datasets are therefore essential.

Beyond imaging, disease-specific antibody testing remains central to diagnosing autoimmune encephalitis and antibody-associated demyelinating diseases (15). However, a proportion of patients remain seronegative, requiring diagnosis based on clinical assessment and non-specific ancillary tests (16). Brain biopsy can increase diagnostic accuracy in selected cases but is used infrequently due to procedural risk (17). Because most AI studies have focused on seropositive cases, incorporating clinical parameters into future models may aid in identifying seronegative neuroimmunological disorders. Given that only a few studies included seronegative patients, there is a clear need for future AI research to focus on developing diagnostic models that can accurately identify seronegative cases.

To improve model performance and reduce variability, methodological strategies such as transfer learning and feature-attribution techniques are recommended, particularly for small datasets (18). Appropriate selection of classification algorithms and rigorous validation approaches, including external testing, can enhance model reliability and reduce bias (19, 20). Pre-trained architectures like ResNet have shown strong generalization (21), and interpretability tools such as Grad-CAM can enhance transparency by highlighting relevant MRI regions (22, 23).

Nevertheless, our work has limitations. Despite extensive screening, relatively few studies evaluated autoimmune encephalitis, ADEM, or MOGAD, limiting conclusions about AI performance in these disorders. External validation remained limited, and our meta-analysis was constrained by the predominance of MRI-based models due to the scarcity of research incorporating other modalities.

Future research should prioritize multicenter datasets, integration of clinical variables, and development of interpretable models to enhance diagnostic precision. While traditional diagnostic tools remain indispensable, AI has strong potential to support and augment neuroimmunological assessment in clinical practice.

## 5 Conclusion

AI approaches show promising potential for differentiating neuroimmunological disorders, with most substantial progress to date in distinguishing MS from NMOSD. Although individual studies often report high performance, our meta-analysis reveals significant heterogeneity driven by differences in study size, dataset composition, and model architecture. Future work should emphasize stronger methodological rigor, consistent external validation, and the integration of clinical and epidemiological variables into diagnostic algorithms. Because antibody testing enables accurate diagnosis for many conditions, AI applications may be particularly valuable for seronegative disorders, where current tools are limited. Overall, our findings offer practical guidance for developing more robust and clinically applicable AI models in neuroimmunology.

## Data Availability

The original contributions presented in the study are included in the article/Supplementary material, further inquiries can be directed to the corresponding author.
